# Investigating Associations Between Prognostic Factors in Gliomas: Unsupervised Multiple Correspondence Analysis

**DOI:** 10.2196/65645

**Published:** 2025-03-12

**Authors:** Maria Eduarda Goes Job, Heidge Fukumasu, Tathiane Maistro Malta, Pedro Luiz Porfirio Xavier

**Affiliations:** 1 Laboratory of Comparative and Translational Oncology, Department of Veterinary Medicine, School of Animal Science and Food Engineering, University of Sao Paulo, Avenida Duque de Caxias, 225, Jardim Elite, Pirassununga, 13.635-900, Brazil, 55 19 9 8367-1821; 2 Cancer Epigenomics Laboratory, Department of Clinical Analysis, Toxicology and Food Sciences, School of Pharmaceutical Sciences of Ribeirao Preto, University of Sao Paulo, Ribeirão Preto, Brazil

**Keywords:** brain tumors, bioinformatics, stemness, multiple correspondence analysis

## Abstract

**Background:**

Multiple correspondence analysis (MCA) is an unsupervised data science methodology that aims to identify and represent associations between categorical variables. Gliomas are an aggressive type of cancer characterized by diverse molecular and clinical features that serve as key prognostic factors. Thus, advanced computational approaches are essential to enhance the analysis and interpretation of the associations between clinical and molecular features in gliomas.

**Objective:**

This study aims to apply MCA to identify associations between glioma prognostic factors and also explore their associations with stemness phenotype.

**Methods:**

Clinical and molecular data from 448 patients with brain tumors were obtained from the Cancer Genome Atlas. The DNA methylation stemness index, derived from DNA methylation patterns, was built using a one-class logistic regression. Associations between variables were evaluated using the *χ*² test with k degrees of freedom, followed by analysis of the adjusted standardized residuals (ASRs >1.96 indicate a significant association between variables). MCA was used to uncover associations between glioma prognostic factors and stemness.

**Results:**

Our analysis revealed significant associations among molecular and clinical characteristics in gliomas. Additionally, we demonstrated the capability of MCA to identify associations between stemness and these prognostic factors. Our results exhibited a strong association between higher DNA methylation stemness index and features related to poorer prognosis such as glioblastoma cancer type (ASR: 8.507), grade 4 (ASR: 8.507), isocitrate dehydrogenase wild type (ASR:15.904), unmethylated MGMT (methylguanine methyltransferase) Promoter (ASR: 9.983), and telomerase reverse transcriptase expression (ASR: 3.351), demonstrating the utility of MCA as an analytical tool for elucidating potential prognostic factors.

**Conclusions:**

MCA is a valuable tool for understanding the complex interdependence of prognostic markers in gliomas. MCA facilitates the exploration of large-scale datasets and enhances the identification of significant associations.

## Introduction

Cancer is a dynamic and heterogeneous disease characterized by several hallmarks controlling and contributing to its development and progression [1]. Cancer research continually generates large scales of data encompassing clinical information, genomic and transcriptomic profiles, prognostic and diagnostic markers, and therapeutic targets [2]. Different approaches have been used to study and associate all these variables to manage this complexity, aiming to reduce the dimensionality and enhance data interpretation and decision-making process. Several features used to study and classify the different types of cancer are based on categorical variables. For instance, the most widely used cancer staging system, TNM, is based on categorical variables, where “T” refers to the size of the primary tumor, “N” refers to the number of lymph nodes affected by cancer, and “M” refers to absence or presence of metastasis [3]. Thus, these biological and clinical variables interact, and their associations can be measured and diagnosticated using statistical tests such as Fisher exact tests and *χ*² tests. However, these approaches could not provide a global and comprehensive picture of the associations between these variables, particularly in datasets with a large number of categorical variables. Therefore, using multivariate and visual analysis methods can significantly improve the analysis and interpretation of associations between clinical and molecular cancer phenotypes.

Brain tumors are a particularly aggressive type of cancer, mostly due to local tissue damage and highly invasive growth. Gliomas, which originate from neuroglial stem cells or progenitor cells, account for 30% of primary brain tumors and 80% of malignant brain tumors [4]. This heterogeneous disease is histologically classified based on anaplasia criteria and predominant cell types such as oligodendroglioma, astrocytoma, and glioblastoma (GBM) [5]. Nevertheless, as further investigation aimed to elucidate the neuropathological mechanisms of gliomas, new variables are considered for characterizing this cancer tumor, leading to reclassifications based on mutational profiles, clinical data, and epigenetic factors [6]. This scenario resulted in different prognosis predictions, diagnosis determination, and treatment responses, contributing to an increasingly complex and stratified understanding of gliomas.

Stemness is a key phenotype of cancer stem cells (CSCs), related to tumor initiation and progression, therapy resistance, and metastasis [7]. CSCs are referred to as a subpopulation of tumor cells able to self-renew and differentiate into distinct cell lineages, enabling those cells to adapt to different environmental situations [8]. Moreover, recent studies have demonstrated associations between stemness features and different histologic classifications or prognostic factors of gliomas [9-11]. Therefore, providing a comprehensive visualization of the associations between clinical features and stemness in brain tumors could be valuable for identifying and determining potential prognostic and therapeutic markers.

Multiple correspondence analysis (MCA) is an unsupervised data science methodology that aims to observe and represent associations between variables disposed in contingency tables, visualizing these associations in a 2D perceptual map. This approach allows for the simultaneous visualization of the relationship between 2 or more characteristics [12]. MCA shares general characteristics, and it is an extension of principal component analysis which is effective in reducing data dimensionality. Thus, MCA can significantly reduce the workload and simplify statistical analysis in healthy research [13]. The results of MCA are typically interpreted in a 2D map, where the relative positions of categories of each variable and their distribution along the dimensions are analyzed. Categories that cluster together and are closer are more likely to be associated, providing key insights into the relationship [14]. Despite its applicability, rigor, and success in other disciplines such as Geography, Epidemiology, and Human Physiology, MCA remains underused in Oncology research and few studies are applying [12,14-16].

By using MCA, we aimed to gain a deeper understanding of the interdependence between stemness and prognostic factors. Our findings revealed associations among molecular and clinical characteristics and prognostic factors, as previously described by the literature [17]. Additionally, we demonstrated the capability of MCA to identify associations between stemness and these prognostic factors. Our results exhibited a strong association between higher stemness index and features related to poorer prognosis, demonstrating the utility of MCA as an analytical tool for elucidating oncological heterogeneity and may also offer a valuable strategy for therapeutic decision-making. This study highlights MCA as a powerful tool for overcoming the barrier of representing the heterogeneity and complexity of cancer variables, particularly in glioma.

## Methods

### Dataset of the Tumor Samples

Clinical and molecular information of a total of 448 patients with brain tumors was obtained from the Cancer Genome Atlas (TCGA). We tailored the dataset to contain only qualitative information, with 12 variables: cancer type, histology, grade, patient’s vital status, IDH (isocitrate dehydrogenase) status, codeletion of chromosomes 1p and 19q arms, MGMT (methylguanine methyltransferase) gene methylation, telomerase reverse transcriptase (TERT) expression, gain of chromosome 19 and 20, chromosome 7 gain and chromosome 10 loss, ATRX (alpha thalassemia/mental retardation syndrome, X-linked) status, and GBM transcriptome subtypes. All categorical variables were selected based on their established role as prognostic factors for brain tumors.

### DNA Methylation Stemness Index

The DNA methylation stemness index (mDNAsi) based on DNA methylation was built using a one-class logistic regression [18] on the pluripotent stem cell samples (embryonic stem cell and induced pluripotent stem cell) from the Progenitor Cell Biology Consortium dataset [19,20]. The algorithm was built and validated as described in the original paper [21]. The mDNAsi was applied in 381 samples from the TCGA database. Malta’s model presented a high correlation among other CSC signatures, providing significant insights into the biological and clinical features of pan-cancer. The workflow to generate the mDNAsi is available in the original paper [21].

### Multiple Correspondence Analysis

MCAs were conducted in the RStudio (version 4.3.1; Posit, PBC) environment using the packages FactoMineR (version 2.11; Institut Agro) [22] and cabootcrs (version 2.1.0; Cranfield University), for creating matrices for MCAs. Contingency tables for the categorical variables were generated, and associations between variables were assessed using a *χ*² test with k degrees of freedom. This was followed by the analysis of the adjusted standardized residuals (ASRs). The *χ*² test evaluates whether the observed associations between categorical variables are nonrandomly associated (*P* value *<*.05). ASRs higher than 1.96 indicate a significant association between variables in the matrix. To perform MCA, the categorical variables should not be randomly associated. To create the perceptual map, inertia was determined as the total *χ*² divided by the number of samples, resulting in the number of associations in the dataset. MCA was performed based on the binary matrices and row and column profiles were determined to demonstrate the influence of each category of variables on the others. Matrices were defined based on the row and column profiles. Eigenvalues were then extracted to represent the number of dimensions that could be captured in the analysis. Finally, the x- and y-axis coordinates of the perceptual map were determined, allowing the category of the variables to be represented and established. In MCA, the spatial distance between categories of different variables reflects their associations. Categories with high coordinates that are close in space are directly associated, while categories presenting high coordinates but opposing coordinates are inversely associated.

### Statistical Analysis

Fisher exact tests and *χ*² tests were performed using RStudio 4.3.1 environment and GraphPad Prism (version 10.3.0; Dotmatics, USA).

### Ethical Considerations

The results published in this paper are in whole based upon data generated by the TCGA Research Network [23]. TCGA Ethics and Policies was originally published by the National Cancer Institute [24].

## Results

### MCA Can Identify Associations Between Different Variables of Gliomas and Patient Vital Status

To determine the suitability of glioma variables for MCA, we first evaluated whether categorical glioma variables were randomly or nonrandomly associated. This involved creating individual contingency tables for each pair of glioma variables (Multimedia Appendices 1-13). Then, we applied *χ*² tests for each contingency table to confirm nonrandom associations (*P* value *<*.05). We also confirmed the associations between categorical variables and patients’ vital status using the Fisher exact test (*P* value *<*.05) (Multimedia Appendix 14). Based on the *χ*² test, the results indicated that only 2 categorical variables, gender and DAXX expression, were randomly associated, suggesting no significant association patterns between these variables and the others. Consequently, gender and DAXX expression were excluded from further analysis.

**Figure 1.**
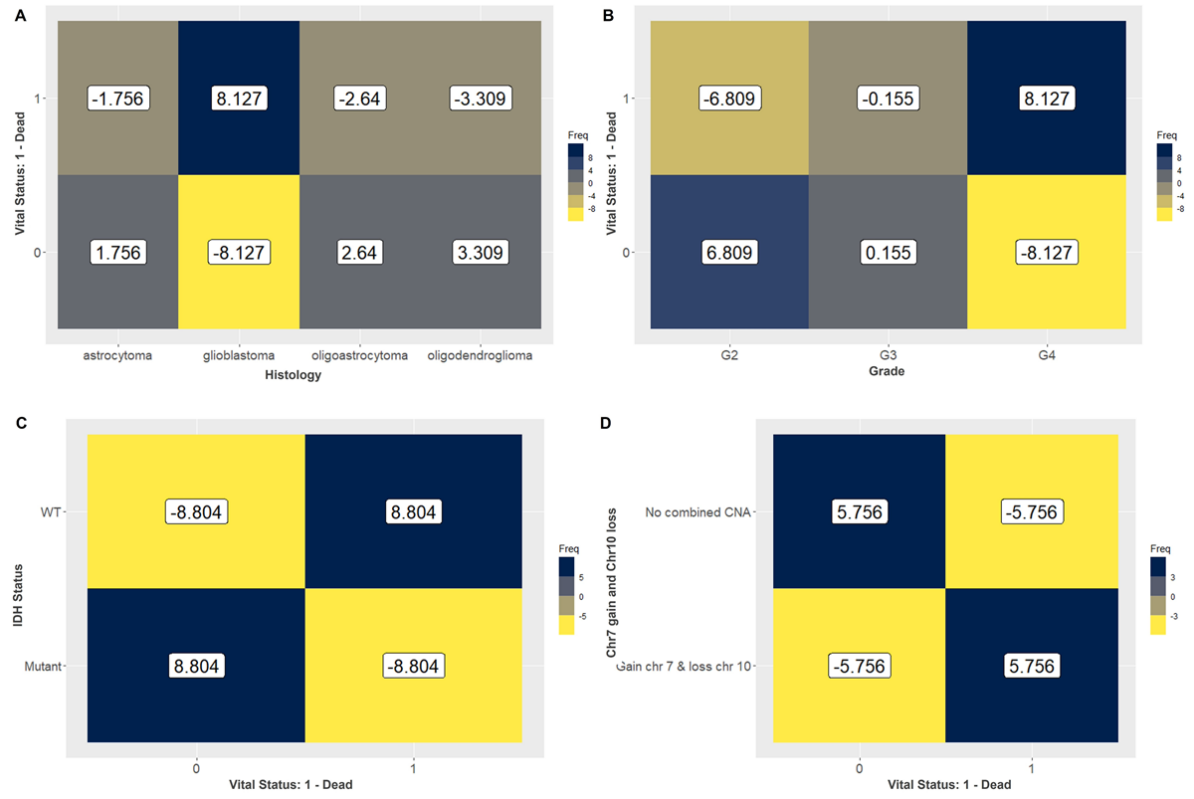
Heatmap exhibiting the values of the adjusted standardized residuals. Categories of variables with values higher than 1.96 are associated. We could observe a strong association of (**A**) glioblastoma (8.127), (**B**) grade 4 (8.127), (**C**) IDH wild type (8.804), and (**D**) Chr7+/Chr10– (5.756) with dead vital status. Favorable prognostic factors including (**A**) oligoastrocytoma and oligodendroglioma, (**B**) grade 2, (**C**) IDH mutant, and (**D**) no combined copy number alterations were associated with alive vital status. Chr7+/Chr10–: chromosome 7 gain and 10 loss; IDH: isocitrate dehydrogenase.

In the subsequent analysis, we observed and measured the strength of associations between the patient vital status (0-alive; 1-dead) and different factors including cancer type, histology, grade, IDH status, 1p19q codeletion, MGMT promoter methylation, gain of chromosome (Chr) 7 and loss of Chr10 (7+/10–), co-gain of Chr19 and Chr20 (19+/20+), TERT expression, ATRX status, and transcriptome subtype, aiming to determine whether MCA could identify associations between prognostic factors for this disease. We used ASRs to assess these associations, considering a category of each variable to be associated with either alive or dead vital status when the ASR values were higher than 1.96. Patients’ vital status classified as dead were associated with poorer prognostics factors such as GBMs, grade 4, IDH wild type, non-codeleted 1p19q, unmethylated MGMT promoter, gain of Chr7 and loss of Chr10, expression of TERT, ATRX wild type, and classical (CL) and mesenchymal (ME) transcriptome subtypes (Table 1). In contrast, patients classified as alive were linked to favorable prognostic variables, including oligoastrocytomas and oligodendrogliomas, grade 2, IDH mutant, codeleted 1p19q, methylated MGMT promoter, absence of combined Chr7+/Chr10– (chromosome 7 gain and 10 loss), lack of TERT expression, ATRX mutant, and the proneural (PN) and neural (NE) transcriptome subtypes (Table 1). Histological classification, grade, IDH status, and Chr7+/Chr10– were the most strongly associated features with patient vital status. These associations were further illustrated in a heatmap (Figure 1A-D).

**Table 1.** Table exhibiting the values of the adjusted standardized residuals. Categories of variables with values higher than 1.96 are considered associated. We could observe a strong association between poorer prognostic factors and dead vital status. In contrast, better prognostic factors were associated with alive vital status.

Glioma variables	Patient vital status		Categories associated with
	Alive	Dead	
Glioblastoma	—^a^	8.127	Dead
Oligoastrocytoma	2.64	**—**	Alive
Oligodendroglioma	3.309	**—**	Alive
Astrocytoma	1.756	**—**	Not associated
Grade 2	6.809	**—**	Alive
Grade 3	0.155	**—**	Not associated
Grade 4	**—**	8.127	Dead
IDH^b^ wild type	**—**	8.804	Dead
IDH mutant	8.804	**—**	Alive
1p/19q codeletion	5.265	**—**	Alive
1p/19q non-codeletion	**—**	5.265	Dead
Methylated MGMT^c^ promoter	5.26	**—**	Alive
Unmethylated MGMT promoter	**—**	5.26	Dead
No combined Chr7+/Chr10–^d^	5.756	**—**	Alive
Chr7+/Chr10–	**—**	5.756	Dead
Not expressed TERT^e^	3.078	**—**	Alive
Expressed TERT	**—**	3.078	Dead
ATRX^f^ mutant	2.311	**—**	Alive
ATRX wild type	**—**	2.311	Dead
Proneural subtype	4.122	**—**	Alive
Neural subtype	3.593	**—**	Alive
Mesenchymal subtype	**—**	4.635	Dead
Classical subtype	**—**	4.852	Dead

a Not applicable.

b IDH: isocitrate dehydrogenase.

c MGMT: methylguanine methyltransferase.

d Chr7+/Chr10–: chromosome 7 gain and 10 loss.

e TERT: telomerase reverse transcriptase.

f ATRX: alpha thalassemia/mental retardation syndrome, X-linked.

Using MCA, we observed that dimension 1 (x-axis) accounted for 33.71% of the variance, while dimension 2 (y-axis) accounted for 14.08%. The inertia (sum of the variances) for these 2 dimensions was 47.79%. The variance of the overall dimensions (17 dimensions) for the combinations of the variables is illustrated in Multimedia Appendix 15. The main idea was to present the percentage of explained variance for each dimension and not the influence of individual variables. The total inertia (sum of the variances) was 1.41.

**Figure 2.**
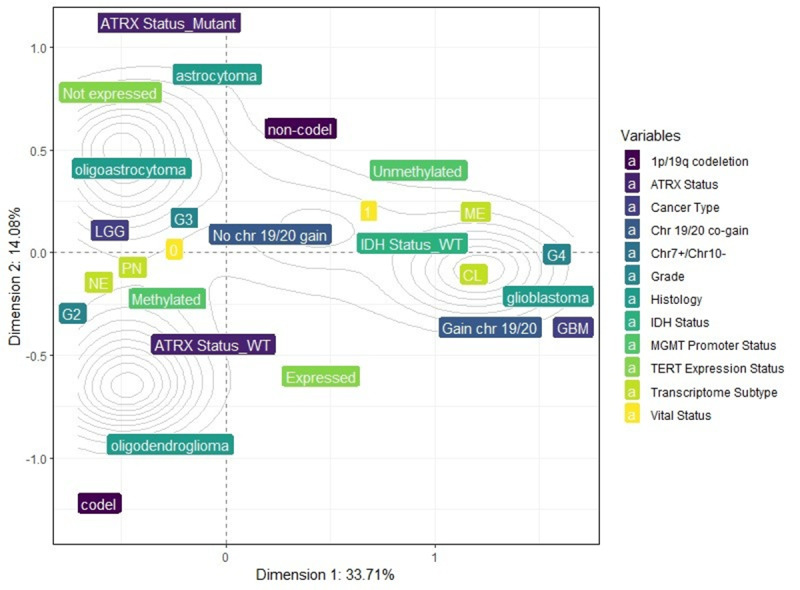
Multiple correspondence analysis (MCA) 2D perceptual map demonstrating the association between the categories of each categorical variable. Categories that are closely clustered are strongly associated with each other. Categories such as glioblastoma, unmethylated MGMT promoter, IDH wild type, chromosome 7 gain and 10 loss (Chr7+/Chr10–), grade 4, glioblastoma ATRX wild type, TERT expression, non-codel 1p.19q, CL and ME transcriptome subtypes are closely associated with dead vital status (1), appearing along the positive x-axis (dimension 1). ATRX: alpha thalassemia/mental retardation syndrome, X-linked; CL: classical; GBM: glioblastoma; IDH: isocitrate dehydrogenase; ME: mesenchymal; MGMT: methylguanine methyltransferase; NE: neural; PN: proneural; TERT: telomerase reverse transcriptase.

The results obtained from the MCA were visualized in a 2D perceptual map (Figure 2), highlighting the associations between the categories of each variable. The coordinates of each category are detailed in Table 2. The perceptual map reveals that categories such as GBM, unmethylated MGMT promoter, IDH wild type, Chr7 gain and Chr10 loss, grade 4, GBM ATRX wild type, TERT expression, non-codel 1p.19q, and CL and ME transcriptome subtypes are closely associated with dead vital status, appearing along the positive x-axis (dimension 1). Conversely, categories like oligoastrocytomas and oligodendrogliomas, grade 2, IDH mutant, codel 1p19q, methylated MGMT promoter, no combined copy number alterations, no expression of TERT, ATRX mutant, and PN and NE transcriptome subtypes are closely associated with alive vital status, appearing along the negative x-axis (dimension 1) (Figure 2).

These findings highlight the utility and capacity of MCA in reducing data dimensionality and demonstrate that, in gliomas, variables interact cohesively. MCA allows us to further visualize these interactions on a global perceptual map, organizing the characteristics into distinct clusters that correspond to different prognostic profiles.

**Table 2.** Coordinates of each category compounding the perceptual map.

Category	Dimension 1 (x-axis)	Dimension 2 (y-axis)
GBM^a^	1.6650830	−0.0896760
Low-grade glioma	−0.4723301	0.0254382
Astrocytoma	−0.2672355	0.9527631
Glioblastoma	1.6650830	−0.0896760
Oligoastrocytoma	−0.5334711	0.3276318
Oligodendroglioma	−0.6011671	−0.9346433
Grade 2	−0.6611308	−0.1971919
Grade 3	−0.2970898	0.2320783
Grade 4	1.6650830	−0.0896760
0-Alive	−0.3185609	−0.0551369
1-Dead	0.7544862	0.1305874
IDH^b^ mutant	−0.6734117	−0.0548104
IDH wild type	1.1888626	0.0967641
1p/19q codel	−0.6877365	−13.034.766
1p/19q non-codel	0.2750946	0.5213906
Methylated	−0.3429710	−0.1087842
Unmethylated	1.0048449	0.3187185
Chr7+/Chr10−^c^	1.4087248	−0.0210234
No combined Chr7+/Chr10−	−0.4205758	0.0062766
Chr 19/20 co-gain	1.4900007	−0.1295089
No Chr 19/20 co-gain	−0.0843397	0.0073307
Expressed TERT^d^	0.3715020	−0.6845760
Not expressed TERT	−0.4690682	0.8643636
ATRX^e^ mutant	−0.6448249	1.0773395
ATRX wild type	0.2693572	−0.4500279
Classical	1.2675815	−0.0217510
Mesenchymal	1.0920361	0.2687642
Neural	−0.5475482	−0.0650952
Proneural	−0.5971662	−0.0604168

a GBM: glioblastoma.

b IDH: isocitrate dehydrogenase.

c Chr7+/Chr10–: chromosome 7 gain and 10 loss.

d TERT: telomerase reverse transcriptase.

e ATRX: Alpha Thalassemia/Mental Retardation Syndrome X-linked.

### MCA Can Associate an Epigenetic Stemness Index (mDNAsi) as a Prognostic Factor in Gliomas

After demonstrating that MCA effectively reduces dimensionality and identifies associations between prognostic factors and clinical data in the glioma database, we proceeded to explore whether MCA could also associate these variables with stemness phenotype. For this analysis, we updated our database by including mDNAsi as a new variable, categorized into low, intermediate, and high levels of stemness. These categories were based on the DNA methylation index related to tumor pathology and clinical outcomes, as previously studied by [21].

First, we evaluated whether the categorical glioma variables were randomly or nonrandomly associated with mDNAsi by creating individual contingency tables for each pair of glioma variables and applying *χ*² tests (Multimedia Appendix 16). We also confirmed the associations between categorical variables using the Fisher exact test (*P* value *<*.05) ( Multimedia Appendix 17). All the variables were found to be suitable for MCA. Then, using ASR values to evaluate the strength of these associations, our results indicated strong associations between high mDNAsi levels and poor prognostic and clinical factors. Higher mDNAsi levels were associated with GBM, IDH wild-type, absence of 1p19q co-deletion, unmethylated MGMT promoter, TERT expression, grade 3 and 4, patient’s vital status as dead, Chr7+/Chr10–, chromosomes 19/20 co-gain, ATRX wildtype and ME and CL transcriptome subtypes (Table 3). Conversely, intermediate and lower levels of mDNAsi were associated with characteristics related to favorable prognosis, including oligodendroglioma, IDH mutant, 1p19q co-deletion, methylation of MGMT promoter, absence of TERT expression, grade 2, patient’s vital status as alive, no combined copy number alteration, absence of chromosomes 19/20 co-gain, ATRX mutant, and PN and NE transcriptome subtypes (Table 3).

**Table 3.** Table exhibiting the values of the adjusted standardized residuals. Categories of variables with values higher than 1.96 are considered associated. We could observe a strong association between poorer prognostic factors and a higher stemness index (DNA methylation stemness index [mDNAsi]). In contrast, better prognostic factors were associated with lower stemness index.

Glioma Variables	mDNAsi			Categories associated with
	Low	Intermediate	High	
Glioblastoma	—^a^	—	8.507	High
Oligoastrocytoma	—	—	—	Not associated
Oligodendroglioma	3.949	—	—	Low
Astrocytoma	—	—	2.832	High
G2	3.279	4.057	—	Low and intermediate
G3	—	—	2.392	High
G4	—	—	8.507	High
IDH^b^ wild type	—	—	15.904	High
IDH mutant	8.743	7.057	—	Low and intermediate
1p/19q codeletion	5.772	2.102	—	Low and intermediate
1p/19q non-codeletion	—	—	7.964	High
Methylated MGMT^c^ promoter	5.944	3.961	—	Low and intermediate
Unmethylated MGMT promoter	—	—	9.983	High
No combined Chr7+/Chr10−^d^	6.436	5.927	—	Low and intermediate
Chr7+/Chr10−	—	—	12.433	High
Not expressed TERT^e^	—	3.216	—	Intermediate
Expressed TERT	—	—	3.351	High
ATRX^f^ mutant	—	3.505	—	Intermediate
ATRX wild type	—	—	4.949	High
Proneural subtype	8.476	—	—	Low
Neural subtype	—	4.218	—	Intermediate
Mesenchymal subtype	—	—	4.771	High
Classical subtype	—	—	10.981	High

a Not applicable.

b IDH: isocitrate dehydrogenase.

c MGMT: methylguanine methyltransferase.

d Chr7+/Chr10–: chromosome 7 gain and 10 loss.

e TERT: telomerase reverse transcriptase.

f ATRX: Alpha Thalassemia/Mental Retardation Syndrome X-linked.

**Figure 3.**
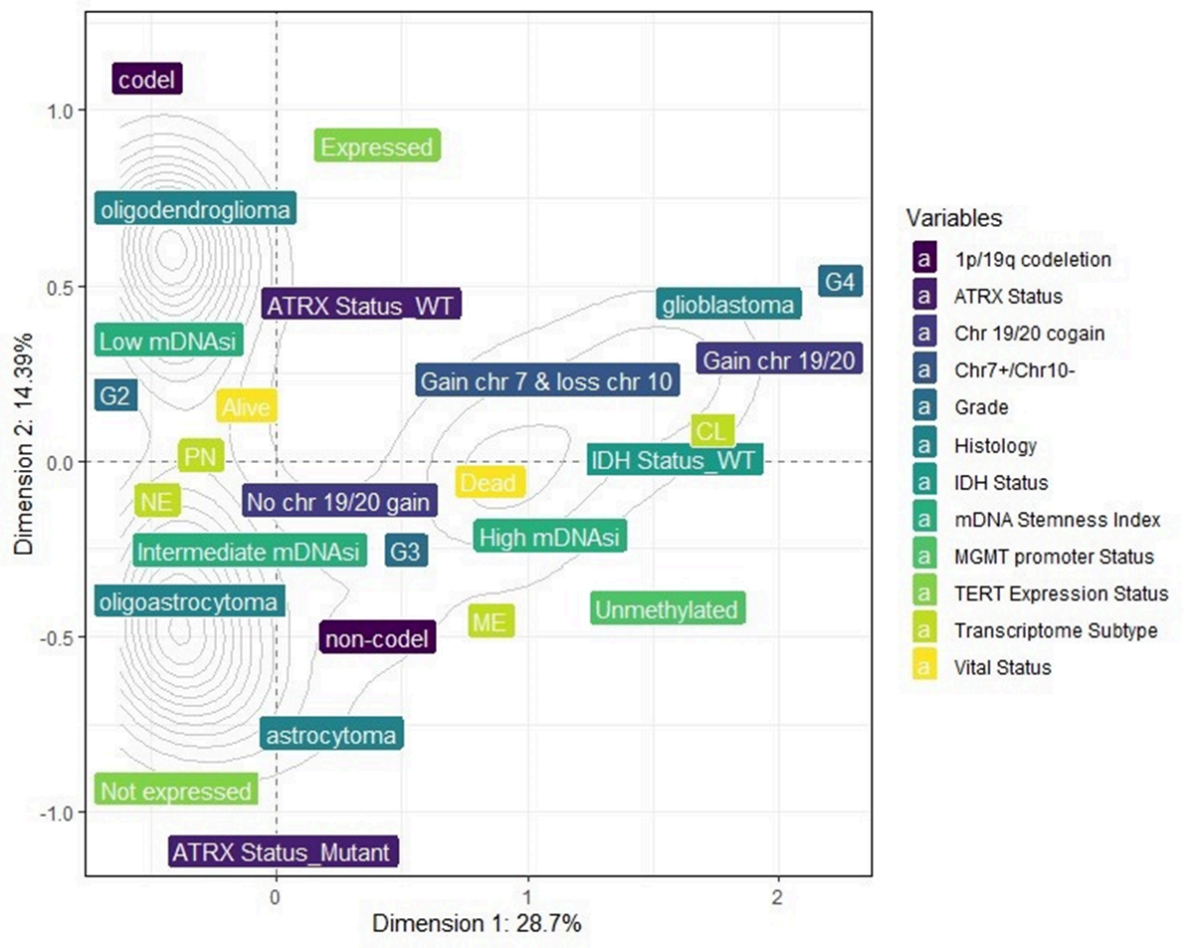
Multiple correspondence analysis (MCA) 2D perceptual map demonstrating the association between the categories of each categorical variable. Categories that are closely clustered are strongly associated with each other. Categories such as glioblastoma, unmethylated MGMT promoter, IDH wild type, chromosome 7 gain and 10 loss (Chr7+/Chr10–), grade 4, glioblastoma ATRX wild type, TERT expression, non-codel 1p.19q, and CL and ME transcriptome subtypes are closely associated with high mDNAsi, appearing along the positive x-axis (dimension 1). ATRX: alpha thalassemia/mental retardation syndrome, X-linked; CL: classical; IDH: isocitrate dehydrogenase; mDNAsi: DNA methylation stemness index; ME: mesenchymal; MGMT: methylguanine methyltransferase; NE: neural; PN: proneural; TERT: telomerase reverse transcriptase.

Using MCA, dimension 1 (x-axis) accounted for 28.7% of the variance, while dimension 2 (y-axis) accounted for 14.39%. The inertia (sum of the variances) for these 2 dimensions was 43.09%. The variance of the overall dimensions (18 dimensions) for the combinations of the variables is illustrated in Multimedia Appendix 18. The total inertia (sum of the variances) was 1.5. The 2D perceptual map exhibited the associations between the categories of each variable (Figure 3). The perceptual map reveals categories such as GBM, unmethylated MGMT promoter, IDH wild type, Chr7 gain and Chr10 loss, grade 4, GBM ATRX wild type, TERT expression, non-codel 1p.19q, and CL and ME transcriptome subtypes are closely associated with high mDNAsi, appearing along the positive x-axis (dimension 1). Conversely, categories like oligoastrocytomas and oligodendrogliomas, grade 2, IDH mutant, codel 1p19q, methylated MGMT promoter, no combined copy number alterations, no expression of TERT, ATRX mutant, and PN and NE transcriptome subtypes are closely associated with alive vital status, appearing along the negative x-axis (dimension 1) (Figure 3).

## Discussion

### Principal Findings

Multiple efforts have been made to explore the diversity of oncologic diseases, with significant contributions from genetics, cell and tissue biology, as well as computational and experimental technologies, providing a wealth of information on cancer manifestations. In the field of glioma research, emerging approaches have sought to clarify tumor pathology and grading through the introduction of novel types and subtypes, as well as by identifying molecular markers and genetic mutations that contribute to predicting diagnosis and prognosis. However, it also results in an accumulation of extensive datasets, presenting challenges in interpretation and visualization regarding the associations between prognostic factors. In this study, we used MCA, an unsupervised data science approach, to establish statistical associations between different qualitative variables of gliomas. This method was able to reduce data dimensionality and represent it on a 2D perceptual map, revealing associations between various established glioma prognostic factors, including histological classification, IDH status, MGMT promoter methylation, and transcriptome subtypes. Furthermore, we associated these clinical and prognostic variables with an epigenetic-based stemness index (mDNAsi), demonstrating that higher stemness levels were associated with poorer prognostic factors, providing a useful tool to associate prognostic markers in brain tumors.

### Comparison to Prior Studies

Several clinical and molecular factors are considered in predicting the prognosis and survival of brain tumors, more specifically for gliomas. Beyond histological classification and tumor grade, genetic and molecular biomarkers have been incorporated as potential prognostic indicators. Thus, we first evaluated the ability of MCA to associate these consolidated prognostic variables with the patient’s vital status. Our findings demonstrate that MCA effectively clusters poor prognostic factors with dead vital status. All these prognostic factors are well consolidated and associated with malignancy of gliomas. IDH mutation represents one of the main prognostic markers for gliomas [25]. It has been identified that one of the mechanisms given by this favorable outcome is the impaired production of nicotinamide adenine dinucleotide phosphate in Krebs cycle caused by IDH1 enzyme mutation that can sensitize tumor cells to chemotherapy and explain the favorable prognosis of patients with IDH mutation [25]. Likewise, co-deletion of 1p19q chromosome arms, especially when combined with other biomarkers such as IDH mutation and TERT expression, has been used as a predictive biomarker and recent studies investigated biological mechanisms to be significantly linked to genes involved in cell division, angiogenesis, and DNA repair responses [26]. Thus, we demonstrated that MCA was able to capture and associate key glioma hallmarks with patients’ vital status, which was applied to different clinical variables.

Subsequently, we applied MCA to explore the association between high stemness levels (mDNAsi) and characteristics related to poor prognosis. Stemness has been considered an important phenotype in glioma malignancy and is potentially associated with CL genetic alterations, such as the gain of chromosome 7. Chromosome 7 harbors some key genes related to stemness, including Epidermal Growth Factor Receptor (EGFR), Mesenchymal-Epithelial Transition Factor (MET), and Homeobox A gene (HOXA). A study of 86 GBMs reported that EGFR amplification occurs with higher probability in samples that have a gain of chromosome 7 (82.1%) compared with those without it (66.7%) [27]. In addition, EGFR amplification is more prevalent in IDH-wildtype diffuse gliomas (66.0%) and GBM (85.5%) [28], which are also associated with poorer prognostic factors, consistent with our findings. High mDNAsi has been previously linked to EGFR mutations [21]. The HOXA and MET loci, also located on chromosome 7, have been implicated in stemness-related pathways. Notably, studies have demonstrated interactions between chromosome 7 gain and the expression of a stem cell-related HOX signature in GBMs [29]. Analysis of the MET gene at 7q31.2 revealed that gain occurs in 47% of primary and 44% of secondary GBMs, suggesting that this genetic alteration contributes to the pathogenesis of both GBM subtypes [30].

Overall, relatively few studies have used MCA to explore associations with cancer phenotypes. Previous studies have applied MCA to different approaches, such as analyzing prognosis low rectal cancer surgery [31], investigating the association between some types of cancer in rural or urban areas [15], examining the association between Traditional Chinese Medicine Syndrome and histopathology of colorectal cancer [32], assessing clinically relevant demographic variables across multiple gastrointestinal cancers [33], and the relationship between types of diagnostic classification in breast cancer [34]. Our study also highlights the utility of MCA in investigating associations within the context of brain tumors. MCA enables the investigation of the pattern among many categorical factors in gliomas, providing a powerful computational approach to identify and test prognostic variables. It was possible to visually and quantitatively represent the associations, which facilitates the identification of distinct patient clusters based on shared prognostic characteristics. Our findings were consistent with previous literature and emphasized stemness as an important phenotype for gliomas.

### Limitations

Our study has inherent limitations. First, as a retrospective analysis of TCGA data, it is subject to selection bias. Second, we associated all the prognostic variables with patients’ vital status, which may not be the most optimal variable for determining prognosis. For the future, we intend to improve our model validating its applicability in other prospective datasets. Third, the absence of therapy data is another limitation of this study. Finally, an intrinsic limitation of MCA is that retaining only 2 or 3 dimensions may not sufficiently capture all the significant features in the data. In our analysis, the percentage of explained inertia was approximately 40%. While there is not an accepted threshold for adequately explained inertia, common guidelines recommend retaining dimensions that represent over 70% of the inertia [35]. However, explained inertia in the range of 40%‐60% is often considered informative, and the interpretability and relevance of the patterns revealed by the dimensions are frequently more important than the exact percentage of inertia explained, especially in a complex heterogeneous disease such as brain tumors [36].

### Conclusion and Future Perspectives

In conclusion, our findings suggest that MCA is a valuable tool for understanding the interdependence between prognostic markers in gliomas. MCA facilitates the exploration of a large-scale dataset and enhances the identification of associations. Considering the advances in computational oncology and the emergence of new oncological features, such as stemness phenotype, incorporating MCA into cancer research as an approach to exploring the complex heterogeneity of the oncologic field becomes a powerful tool for simplifying data management. It contributes to researchers statistically identifying associations between variables within extensive databases and improves the visual representation, leading to a deeper understanding of cancer findings.

## Supplementary material

10.2196/65645 Multimedia Appendix 1 Individual contingency tables for cancer type.

10.2196/65645 Multimedia Appendix 2 Individual contingency tables for histology.

10.2196/65645 Multimedia Appendix 3 Individual contingency tables for grade.

10.2196/65645 Multimedia Appendix 4 Individual contingency tables for gender.

10.2196/65645 Multimedia Appendix 5 Individual contingency tables for vital status.

10.2196/65645 Multimedia Appendix 6 Individual contingency tables for IDH (isocitrate dehydrogenase) status.

10.2196/65645 Multimedia Appendix 7 Individual contingency tables for X1p.19q.codeletion.

10.2196/65645 Multimedia Appendix 8 Individual contingency tables for MGMT (methylguanine methyltransferase) promoter.

10.2196/65645 Multimedia Appendix 9 Individual contingency tables for Chr 7 gain and Chr 10 loss.

10.2196/65645 Multimedia Appendix 10 Individual contingency tables for Chr 19/20 co-gain.

10.2196/65645 Multimedia Appendix 11 Individual contingency tables for TERT (telomerase reverse transcriptase) expression status.

10.2196/65645 Multimedia Appendix 12 Individual contingency tables for ATRX (Alpha Thalassemia/Mental Retardation Syndrome X-linkedalpha thalassemia/mental retardation syndrome, X-linked) status.

10.2196/65645 Multimedia Appendix 13 Individual contingency tables for DAXX status.

10.2196/65645 Multimedia Appendix 14 Fisher exact test and *χ*² test for vital status × glioma prognostic factors.

10.2196/65645 Multimedia Appendix 15 Percentage of explained variances of the overall (17) dimensions.

10.2196/65645 Multimedia Appendix 16 Individual contingency table for mDNAsi.

10.2196/65645 Multimedia Appendix 17 Fisher exact test and *χ*² test for mDNAsi (DNA methylation stemness index) × glioma prognostic factors.

10.2196/65645 Multimedia Appendix 18 Percentage of explained variances of the overall (18) dimensions.

## Abbreviations

ASR: adjusted standardized residual
ATRX: alpha thalassemia/mental retardation syndrome, X-linked
Chr: chromosome
Chr7+/Chr10–: chromosome 7 gain and 10 loss
CL: classical
CSC: cancer stem cell
GMB: glioblastoma
IDH: isocitrate dehydrogenase
MCA: multiple correspondence analysis
mDNAsi: DNA methylation stemness index
ME: mesenchymal
MGMT: methylguanine methyltransferase
NE: neural
PR: proneural
TCGA: the Cancer Genome Atlas
TERT: telomerase reverse transcriptase

## References

[1] Hanahan D (2022). Hallmarks of cancer: new dimensions. Cancer Discov.

[2] Dagogo-Jack I, Shaw AT (2018). Tumour heterogeneity and resistance to cancer therapies. Nat Rev Clin Oncol.

[3] Brierley J, O’Sullivan B, Asamura H (2019). Global consultation on cancer staging: promoting consistent understanding and use. Nat Rev Clin Oncol.

[4] Weller M, Wick W, Aldape K (2015). Glioma. Nat Rev Dis Primers.

[5] Louis DN, Ohgaki H, Wiestler OD (2007). The 2007 WHO classification of tumours of the central nervous system. Acta Neuropathol.

[6] Louis DN, Perry A, Wesseling P (2021). The 2021 WHO classification of tumors of the central nervous system: a summary. Neuro Oncol.

[7] Ayob AZ, Ramasamy TS (2018). Cancer stem cells as key drivers of tumour progression. J Biomed Sci.

[8] Batlle E, Clevers H (2017). Cancer stem cells revisited. Nat Med.

[9] Wang Q, Hu B, Hu X (2017). Tumor evolution of glioma-intrinsic gene expression subtypes associates with immunological changes in the microenvironment. Cancer Cell.

[10] Ortensi B, Setti M, Osti D, Pelicci G (2013). Cancer stem cell contribution to glioblastoma invasiveness. Stem Cell Res Ther.

[11] Tan J, Zhu H, Tang G (2021). Molecular subtypes based on the stemness index predict prognosis in glioma patients. Front Genet.

[12] Sourial N, Wolfson C, Zhu B (2010). Correspondence analysis is a useful tool to uncover the relationships among categorical variables. J Clin Epidemiol.

[13] Li BH, Sun ZQ, Dong SF (2010). Correspondence analysis and its application in oncology. Commun Stat Theory Methods.

[14] Costa PS, Santos NC, Cunha P, Cotter J, Sousa N (2013). The use of multiple correspondence analysis to explore associations between categories of qualitative variables in healthy ageing. J Aging Res.

[15] Florensa D, Godoy P, Mateo J (2021). The use of multiple correspondence analysis to explore associations between categories of qualitative variables and cancer incidence. IEEE J Biomed Health Inform.

[16] van Horn A, Weitz CA, Olszowy KM (2019). Using multiple correspondence analysis to identify behaviour patterns associated with overweight and obesity in Vanuatu adults. Public Health Nutr.

[17] Śledzińska P, Bebyn MG, Furtak J, Kowalewski J, Lewandowska MA (2021). Prognostic and predictive biomarkers in gliomas. Int J Mol Sci.

[18] Sokolov A, Paull EO, Stuart JM ONE-class detection of cell states in tumor subtypes.

[19] Salomonis N, Dexheimer PJ, Omberg L (2016). Integrated genomic analysis of diverse induced pluripotent stem cells from the progenitor cell biology consortium. Stem Cell Rep.

[20] Daily K, Ho Sui SJ, Schriml LM (2017). Molecular, phenotypic, and sample-associated data to describe pluripotent stem cell lines and derivatives. Sci Data.

[21] Malta TM, Sokolov A, Gentles AJ (2018). Machine learning identifies stemness features associated with oncogenic dedifferentiation. Cell.

[22] Lê S, Josse J, Husson F (2008). FactoMineR: an R package for multivariate analysis. J Stat Softw.

[23] The Cancer Genome Atlas program (TCGA). Center for Cancer Genomics.

[24] The Cancer Genome Atlas program. National Cancer Institute.

[25] Bleeker FE, Atai NA, Lamba S (2010). The prognostic IDH1( R132 ) mutation is associated with reduced NADP+-dependent IDH activity in glioblastoma. Acta Neuropathol.

[26] Chai RC, Zhang KN, Chang YZ (2019). Systematically characterize the clinical and biological significances of 1p19q genes in 1p/19q non-codeletion glioma. Carcinogenesis.

[27] McNulty SN, Cottrell CE, Vigh-Conrad KA (2019). Beyond sequence variation: assessment of copy number variation in adult glioblastoma through targeted tumor somatic profiling. Hum Pathol.

[28] Wang H, Zhang X, Liu J (2024). Clinical roles of EGFR amplification in diffuse gliomas: a real-world study using the 2021 WHO classification of CNS tumors. Front Neurosci.

[29] Kurscheid S, Bady P, Sciuscio D (2015). Chromosome 7 gain and DNA hypermethylation at the HOXA10 locus are associated with expression of a stem cell related HOX-signature in glioblastoma. Genome Biol.

[30] Pierscianek D, Kim YH, Motomura K (2013). MET gain in diffuse astrocytomas is associated with poorer outcome. Brain Pathol.

[31] Mancini R, Pattaro G, Diodoro MG (2018). Tumor regression grade after neoadjuvant chemoradiation and surgery for low rectal cancer evaluated by multiple correspondence analysis: ten years as minimum follow-up. Clin Colorectal Cancer.

[32] Wu T, Zhang S, Guo S (2015). Correspondence analysis between traditional Chinese medicine (TCM) syndrome differentiation and histopathology in colorectal cancer. Eur J Integr Med.

[33] Kramer RJ, Rhodin KE, Therien A (2024). Unsupervised clustering using multiple correspondence analysis reveals clinically-relevant demographic variables across multiple gastrointestinal cancers. Surgical Oncology Insight.

[34] Nadjib Bustan M, Arif Tiro M, Annas S (2019). Correspondence analysis of breast cancer diagnosis classification. J Phys Conf Ser.

[35] Higgs NT (1991). Practical and innovative uses of correspondence analysis. R Stat Soc Ser D (The Statistician).

[36] Husson F, Lê S, Pagès J (2011). Exploratory Multivariate Analysis by Example Using R.

[37] Machine learning identifies stemness features associated with oncogenic dedifferentiation. National Cancer Institute.

[38] PanCanStem: reproducing mrnasi from PMID: 29625051. GitHub.

