# Supplementary figures and images for: Investigating Associations Between Prognostic Factors in Gliomas: Unsupervised Multiple Correspondence Analysis
Source: JMIR Bioinform Biotechnol. 2025 Mar 12;6:e65645. doi: 10.2196/65645 (PMC11922494; doi:10.2196/65645)

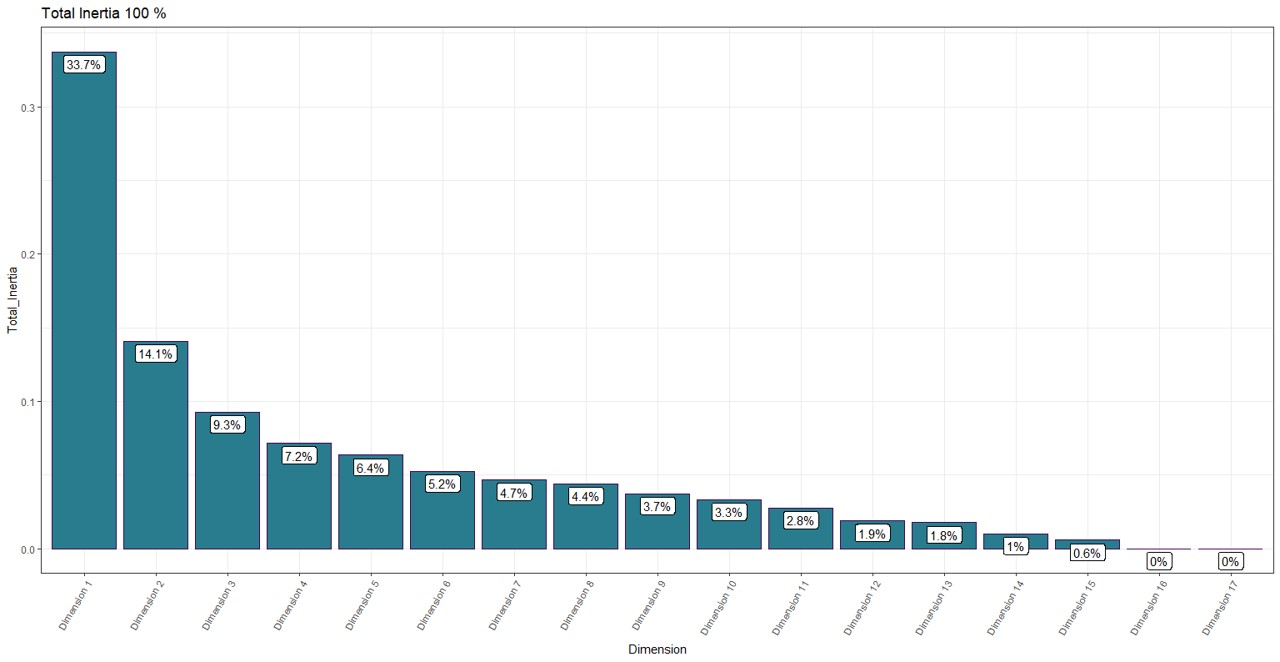

Supplement: Multimedia Appendix 15 [file bioinform-v6-e65645-s015.png]

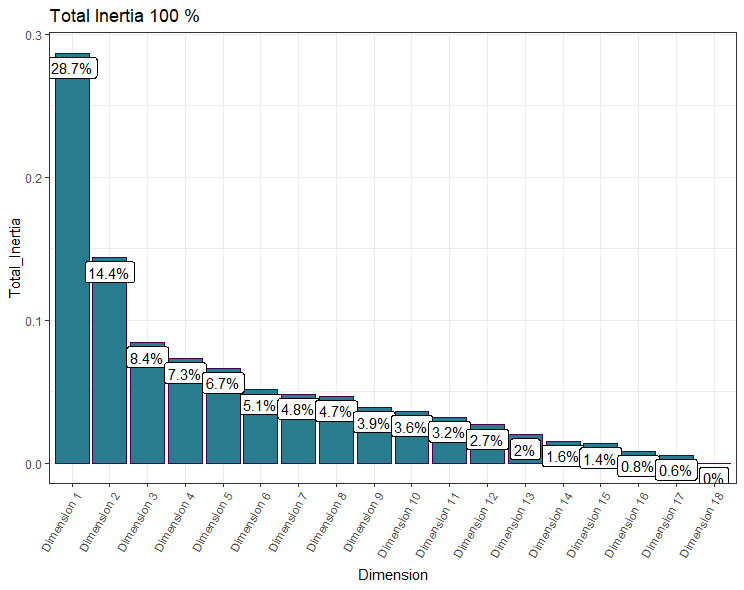

Supplement: Multimedia Appendix 18 [file bioinform-v6-e65645-s018.png]
